# A New Position Measurement System Using a Motion-Capture Camera for Wind Tunnel Tests

**DOI:** 10.3390/s130912329

**Published:** 2013-09-13

**Authors:** Hyo Seon Park, Ji Young Kim, Jin Gi Kim, Se Woon Choi, Yousok Kim

**Affiliations:** 1 Department of Architectural Engineering, Yonsei University, 134 Shinchon-dong, Seoul 110-732, Korea; E-Mails: hspark@yonsei.ac.kr (H.S.P.); cctomoyo@yonsei.ac.kr (J.G.K.); 2 Disaster Prevention Research Team, Daewoo Institute of Construction Technology, 60, Songjuk-dong, Jangan-gu, Kyounggi-do 440-800, Korea; E-Mail: jiyoung.kim@daewooenc.com; 3 Center for Structural Health Care Technology in Buildings, Yonsei University, 134 Shinchon-dong, Seoul 110-732, *Korea*; E-Mail: watercloud@yonsei.ac.kr

**Keywords:** motion-capture camera, wind tunnel test, 3D displacement measurement, vision-based monitoring

## Abstract

Considering the characteristics of wind tunnel tests, a position measurement system that can minimize the effects on the flow of simulated wind must be established. In this study, a motion-capture camera was used to measure the displacement responses of structures in a wind tunnel test, and the applicability of the system was tested. A motion-capture system (MCS) could output 3D coordinates using two-dimensional image coordinates obtained from the camera. Furthermore, this remote sensing system had some flexibility regarding lab installation because of its ability to measure at relatively long distances from the target structures. In this study, we performed wind tunnel tests on a pylon specimen and compared the measured responses of the MCS with the displacements measured with a laser displacement sensor (LDS). The results of the comparison revealed that the time-history displacement measurements from the MCS slightly exceeded those of the LDS. In addition, we confirmed the measuring reliability of the MCS by identifying the dynamic properties (natural frequency, damping ratio, and mode shape) of the test specimen using system identification methods (frequency domain decomposition, FDD). By comparing the mode shape obtained using the aforementioned methods with that obtained using the LDS, we also confirmed that the MCS could construct a more accurate mode shape (bending-deflection mode shape) with the 3D measurements.

## 1. Introduction

To more accurately evaluate wind loads, each country's design code, including ASCE standard 7, requires or allows wind tunnel tests, which have gained recognition as an indispensable test in the field of civil engineering [1,2]. Wind tunnel test equipment, which simulates the actual wind flow, have been developed and modified by comparative analyses using the results of wind tunnel tests and the measured results of actual buildings [3,4]. Moreover, the development of a measurement system has allowed wind tunnel test equipment to measure the actual loads on buildings more accurately.

The high-frequency base balance (HFBB, [5,6]) and synchronous multi-pressure sensing system (SM-PSS, [7,8]) are the typical measurement systems used in wind tunnel tests. The advantage of the HFBB test is that it can provide the base moments and base shear of a target structure subjected to wind loads at a relatively low cost and over a short period of time; however, its limitation is that it cannot provide the vertical profile of the moments and shear force. In contrast, SM-PSS tests can output the vertical profile of the moments and shear force accurately as well as all of the information that can be obtained using the HFBB test. However, these measurement systems are primarily used to evaluate wind loads and are limited to the calculation of the root mean square (RMS) displacement through a spectrum obtained from the aforementioned methods [9]. A measurement method for the deformation of target structures has not been established thus far. Therefore, measuring and evaluating indicators, such as the drift of structures by wind loads, are essential to estimate the safety of buildings and to evaluate the structural performance of a building.

As displacement measurements have become important in wind tunnel tests, the conventional method used to calculate the displacement responses consists of evaluating the double integral of the acceleration data [10,11]. However, this method, as many research results have indicated, has several accuracy problems [12]. Furthermore, the strain value observed from the structural members can be used to evaluate displacement [13,14]; however, many strain measurements are required for an accurate evaluation of the structural behavior.

In addition to the reliability issue associated with conventional measurement devices, instruments such as strain gauges and accelerometers must be installed on the target structures with cables. This installation presents a problem because these wiring systems may affect the wind flow. Installing a wired measurement system can be especially difficult, depending on the profile or shape of the target structures. Thus, the measurement instruments and wiring systems should be shaped and placed such that they do not affect the dynamic characteristics of the target structures and simulated wind flow; establishing a wireless remote sensing system to measure the displacement in wind tunnel tests is necessary.

Remote sensing techniques, such as Light Detection And Ranging (LIDAR), are currently applied to geologic surveys [15], urban features [16,17], and forestry [18]. In addition to LIDAR, Sound Detection And Ranging (SODAR) has been increasingly employed in wind energy research to infer wind speed and direction [19]. Furthermore, a terrestrial laser scanner has been used to monitor structural deformation [20] and landslide displacement [21,22], which are directly related to safety evaluations. However, these systems cannot measure the dynamic displacement of a structure that is induced by dynamic loading, such as wind load. Vision-based displacement measurement systems that use a digital charged coupled device (CCD) camera and image processing techniques [23,24] have also been adopted in the field of structural health monitoring. Although these remote sensing systems have quite accurately measured displacement (0.1−1 mm), their application has also exhibited several shortcomings and problems [20].

This research used a motion-capture camera, which has primarily been used and was developed for human motion science, to measure the displacement responses of structures in a wind tunnel test. The motion-capture system (MCS) can be installed relatively far from the target structure with a sufficiently long reference distance as a remote sensing system for position measurements with a highly accurate and credible measurement performance. Furthermore, in contrast to the existing vision-based displacement measurement systems that capture orthogonal images, the MCS can perform 3D displacement measurements from images on the same plane using at least two cameras. Therefore, the MCS has relatively few installment-related spatial limitations and can measure the deformation of structures induced by cross-wind and along-wind forces. Moreover, the calculated acceleration, which is computed by taking the second derivative of the measured displacement data, is more reliable than the displacement computed by evaluating the double integral of acceleration. Thus, we can simultaneously attain all of the information (displacement, velocity, acceleration, and loads) regarding the structure's response if a position measurement system that uses motion capture is used along with the existing measuring systems (e.g., HFBB and SM-PSS) to calculate the wind loads.

In this research, we performed wind tunnel tests on a pylon structure to assess the applicability and efficiency of the proposed system as a remote sensing system to measure position. We compared the displacement measured using the MCS to that obtained using a laser displacement sensor (LDS). We also examined the credibility and applicability of the displacement measurement obtained during wind tunnel tests by observing the system identification of the target structures through the measurement data.

## 2. Motion-Capture System

### 2.1. Characteristics and Composition of the MCS

The MCS applied in this study uses optical motion capture. The MCS's underlying principle of measurement is the calculation of 3D space coordinates via two-dimensional coordinates obtained by capturing a marker that is attached to a measurement target with at least two cameras. The MCS allows for high-speed photography, incurs no data loss, and is not subject to limitations of the target movement, although it requires a process of creating camera information (position, properties) and virtual coordinates in advance through calibration. Optical motion capture can be categorized into two types depending on how the camera recognizes the marker. First, the passive marker method tracks the position of the marker by reflecting light or infrared light from it with a camera. Second, the active marker method tracks the position of the marker by directly shining light on the marker.

**Figure 1.**
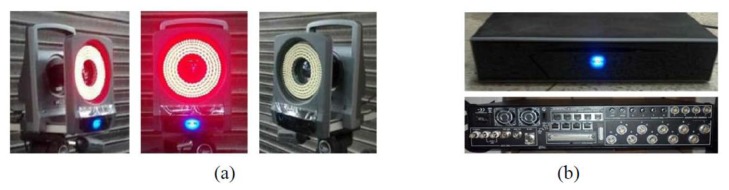
Motion-capture system. (**a**) Camera; (**b**) Vicon giganet.

The MCS (Vicon Motion System, Los Angeles, CA, USA [25]) used in this research consisted of a motion capture camera (Model: T160), passive markers, Vicon giganet, and a PC (post-processing program, NEXUX in the Vicon Motion System), as shown in Figure 1. The markers were attached to target locations to detect and track movements, and a strobe was installed on the camera, which generates infrared light and receives the reflected infrared light from the markers to estimate the location. The Vicon giganet, which connected the camera and computer, synchronized the multiple measurement cameras, obtained the measured data, and transported the data to the PC. The PC generated the 3D coordinates of the targets (markers) using the data measured with the post-processing program, followed by an operation that yielded the final displacement data.

### 2.2. Measurement Principles and Methods

**Figure 2.**
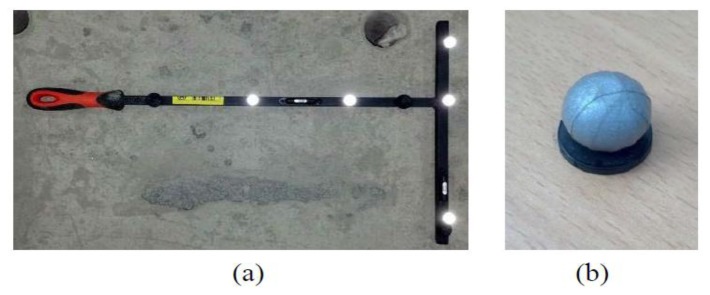
T-shaped wand with five markers. (**a**) Wand; (**b**) Marker.

In the MCS, the position of the target object using markers in 3D coordinates can be obtained as follows:

1. Select the number of cameras (at least two) and the lens depending on the magnitude and range of the behavior of the measurement target.
2. Attach the markers to the selected measurement locations.
3. Perform a calibration to create the virtual 3D space coordinates using a wand with markers (Figure 2).
4. Calculate the relationship between the virtual 3D coordinate (global coordinate) and the coordinates of the measurement target (local coordinate).
5. Obtain the two-dimensional image coordinates in the actual measurement.
6. Perform a 3D reconstruction based on the results obtained from steps 3, 4, and 5.

#### 2.2.1. Coordinate Transformations

Three types of coordinate systems (*i.e.*, image, camera, and global coordinates) are involved in transforming the 2D image coordinates obtained from the camera to 3D global coordinate information. At first, we can represent {*x*} and {*y*} as a 3D point with homogeneous coordinates and as an image for this point in the camera coordinate, respectively. Furthermore, the relationship between {*x*} and {*y*} can be expressed by Equation (1) using the intrinsic camera matrix, which is established from the camera properties (focal length) and origin, *i.e.*, the image coordinate {*y*} is represented by the camera coordinate:

(1) $$\left\{\begin{array}{c} y_{1}\\ y_{2}\\ 1 \end{array}\right\}=[c]\left[I|0\right]\left\{\begin{array}{c} x_{1}\\ x_{2}\\ x_{3}\\ 1 \end{array}\right\}=\left[\begin{array}{ccc} f_{1} & 0 & c_{1}\\ 0 & f_{2} & c_{2}\\ 0 & 0 & 1 \end{array}\right]\left[\begin{array}{cccc} 1 & 0 & 0 & 0\\ 0 & 1 & 0 & 0\\ 0 & 0 & 1 & 0 \end{array}\right]\left\{\begin{array}{c} x_{1}\\ x_{2}\\ x_{3}\\ 1 \end{array}\right\}$$

where [*c*] is the intrinsic camera matrix, *f*_1_ and *f*_2_ are the focal lengths of the cameras, and *c*_1_ and *c*_2_ are the principal points, which are usually at the image center.

Furthermore, the image coordinate {*y*} obtained from the camera should be transformed to the global (world) coordinate{*X*}, which can represent the position of the target structure. Therefore, an additional relationship between the camera coordinate and global coordinate is needed and can be represented by Equation (2) using the extrinsic camera matrix, which describes the camera's location in global coordinates and consists of rigid body transformation components in the rotational (R) and translational (T) directions:

(2) $$\left\{\begin{array}{c} x_{1}\\ x_{2}\\ x_{3}\\ 1 \end{array}\right\}={\left[R|T\right]}_{4\times 4}\left\{\begin{array}{c} X_{1}\\ X_{2}\\ X_{3}\\ 1 \end{array}\right\}=\left[\begin{array}{cccc} r11 & r12 & r13 & t1\\ r21 & r22 & r23 & t2\\ r31 & r32 & r33 & t3\\ 0 & 0 & 0 & 1 \end{array}\right]\left\{\begin{array}{c} X_{1}\\ X_{2}\\ X_{3}\\ 1 \end{array}\right\}$$

#### 2.2.2. Calibration of MCS

The motion-capture camera runs through a two-step calibration process (dynamic calibration and static calibration) to create both the intrinsic and extrinsic camera matrices. The calibration modifies the coordinate variables so that information obtained from the camera after measuring the T-shaped wand with markers (Figure 2), which has dimensions that are exactly known, agrees with the wand standards.

**Figure 3.**
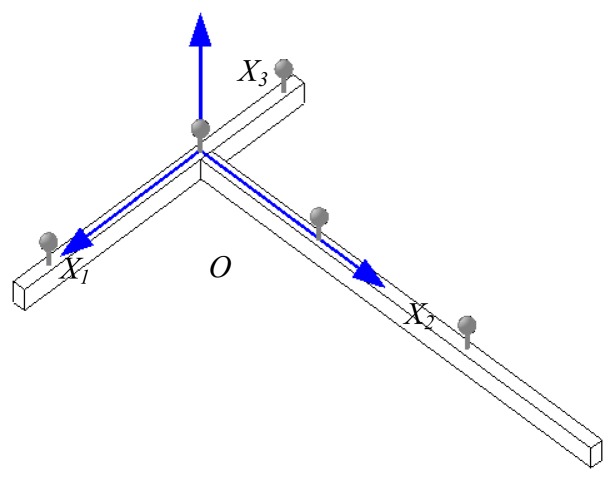
Global coordinates.

First, dynamic calibration (volume calibration) determines the coordinates of the motion-capture cameras, with each camera reading markers (exact distance between the markers) that are attached to the wand and determines the camera space coordinates. During dynamic calibration, a wand with markers is swayed in the air for each camera to capture all the markers, enabling the intrinsic camera matrix that contains the information about the camera and lens variables to then be obtained. After determining the location and orientation of the cameras, a static calibration (set volume origin) was performed that also used the T-shaped wand. During the static calibration, the wand was placed on the ground near the monitoring target. As a result, each axis of the T-shaped wand (*i.e.*, short and long directions) and the orthogonal axes about the wand plane formed the global coordinate (*X*_1_, *X*_2_, and *X*_3_) (Figure 3). Throughout these processes, the relationship between the image coordinates and the global (world or wand) coordinate could be expressed by the camera matrix, which is composed of the intrinsic and extrinsic matrices, as shown in Equation (3):

(3) $$\left\{\begin{array}{c} y_{1}\\ y_{2}\\ 1 \end{array}\right\}=[c]\left[I|0\right]\left[R|T\right]\left\{\begin{array}{c} X_{1}\\ X_{2}\\ X_{3}\\ 1 \end{array}\right\}$$

The image coordinates of the marker calculated by the camera are the result of a perspective projection of the camera coordinates and therefore are two-dimensional coordinates without depth information. To determine the 3D coordinates, at least two cameras are required to perform the measurement and calibration.

However, these 3D coordinates are still slightly off the actual marker coordinates because of a wide range of factors (lens distortion, temperature, humidity, and the dispersed effect by reflected infrared light passing through the lenses). Furthermore, digital cameras require a correction process to obtain more accurate coordinates because the location of the light focused on the sensor is divided into pixels. In this study, the calibration and 3D image reconstruction were performed by the Vicon motion system (NEXUX), which is based on a nonlinear correction method [26].

A filtering process is also performed before generating the final measured data to correct for the distortion of the measured data due to noise and unsatisfactory calibration. The software (NEXUX) for the data post-processing offered by Vicon uses a generalized cross-validation spline smoother (GCVSPS) algorithm, which is generally called the Woltring filter [27,28].

The salient feature of the MCS is its ability to evaluate the relationship between the image coordinates and global (world) coordinates via the calibration process by using a standard wand with markers, the size and position of which are precisely predefined. This process yields 3D coordinate information from the image coordinate information obtained from at least two cameras without any constraint conditions for the camera arrangement (e.g., principal and orthogonal directions). However, the reliability of the MCS measurements strongly depends on the accuracy of calibration, which should be performed before the main monitoring of the target structure. Furthermore, the precision level of the measured displacement should be evaluated from a direct measurement before each test and subsequently varied according to the measurement conditions because the precision of the motion-capture equipment is determined based on the camera pixels, view angle of the lenses, size of the markers, distances between the camera and markers, and testing environment.

**Figure 4.**
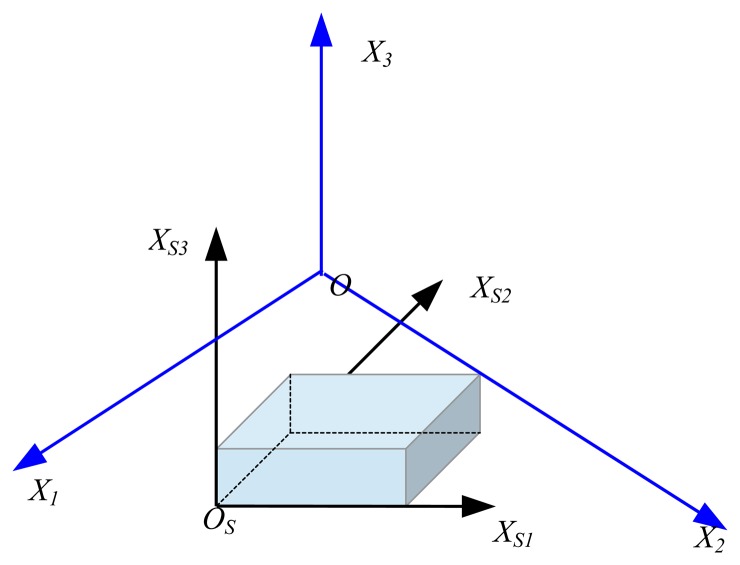
Relationship between the local and global coordinates.

In the actual measurements, the local (structural) coordinates (*X_S1_*, *X_S2_* and *X_S3_*) and global coordinates (*X_1_*, *X_2_*, and *X_3_*) obtained from the wand calibration do not agree, as shown in Figure 4, although we intend to place the wand axes in accordance with the axes of the target structure. Therefore, the relationship between the local coordinates of the target object and the global coordinates should be established and converted through the measured results. In this research, we performed a transformation process before performing the actual measurement to equate the 3D coordinate (global axis) obtained from the wand measurement with the measured results (local axis) obtained from the markers on the measurement target.

## 3. Test Setup

Wind tunnel tests can be categorized as either aeroelastic [29,30] or aerodynamic [31,32] depending on the effect of the target structure's responses to the wind on the wind flow. Therefore, the characteristics of the structure, such as stiffness and mass, should be designed according to the similitude requirement [33] to accurately replicate the prototype building and construct a test model using an aeroelastic method. In contrast, target structures for aerodynamic methods have a rigid body type. Thus, aerodynamic methods allow the wind loads to be calculated relatively rapidly using basic information about the floor plan/elevation of the buildings in the design phase, whereas aeroelastic methods require a large amount of time for the entire process, from designing a test model to obtaining the test results. Aerodynamic models can be applied to most buildings; however, aeroelastic methods should nonetheless be applied to lightweight towers and buildings with large open areas because of the cross-wind effect caused by vortex shedding. In particular, slender high-rise buildings developed from a structural system and construction materials require aeroelastic methods to evaluate their precise wind loads. Thus, aeroelastic methods require accurate measurements to design a prototype structure and predict the dynamic behavior of the target structure.

The primary purpose of this research was to examine the applicability and efficiency of the MCS as a displacement measurement system for wind tunnel tests, and therefore we excluded precise modeling for the prototype structures and wind flow from the test conditions. Instead, we focused on the comparison of the measured data obtained from the MCS with that from the conventional displacement measurement device. We used a pylon structure as the target structure and uniform flow as the input wind flow for our wind tunnel test.

**Figure 5.**
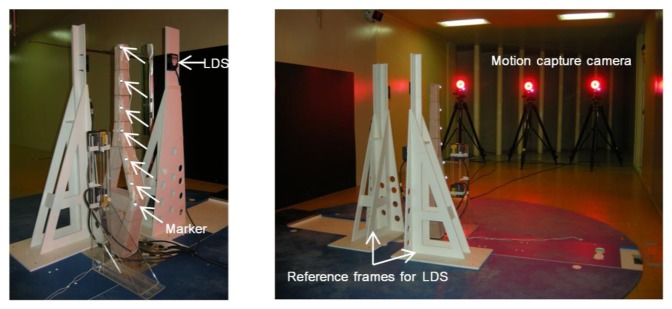
Specimen setup.

**Figure 6.**
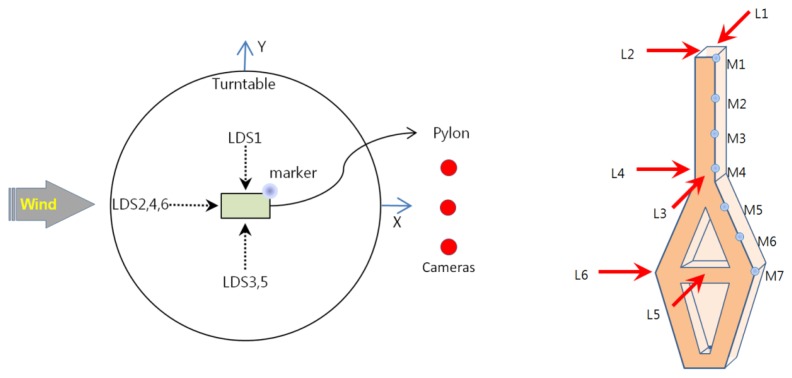
Instrumentation.

As shown in Figure 5 three motion-capture cameras were installed approximately 2.5 m away from the target. As seen in the figure, although the three cameras were located on the same plane, they offered 3D coordinates, which reduced the limitations on the installation areas compared with the existing vision-based displacement systems, for which cameras should be installed orthogonally to obtain 3D coordinates. Consequently, the measurement equipment could be arranged to minimize the effect on the simulated wind flow. Seven passive markers (14 mm in diameter) were attached in the direction of the height of the pylons to perform the measurements (Figure 6). The marker sizes available on the market [25] ranged from 3 mm to 25 mm. Therefore, smaller markers can be used to reduce the effect of the marker itself on the wind flow or structural performance in actual wind tunnel tests in which the aeroelastic method is employed.

To compare the test results obtained using the MCS with the displacements measured by the conventional displacement measurement device, we also installed LDSs (IL300, Keyence, Itasca, IL, USA [34]). We performed measurements at a total of six locations, which were three locations each on the x- and y-axes in the direction of height, as shown in Figure 6. We set up the measurement point of the LDS and the height of the markers on the specimen to be the same for a direct comparison. During the test, measurements from all instruments were performed at a sampling rate of 100 Hz.

In the case of the LDS, the reference distance and measurement range were 300 mm and ±160 mm, respectively, and installing the reference frame for the LDS near the target object was imperative. We installed the reference frame separately from the LDSs with respect to the two considered horizontal directions (Figure 5). As mentioned previously, because simulating accurate wind flow was not considered in this test, we could neglect the effect of the reference frame on the wind flow. However, when such measurement systems (e.g., reference frames for LDS installment) are used for actual wind tunnel tests to simulate accurate wind flow, we can easily see that the measurement frames will affect and change the simulated wind flow.

## 4. Test Results

### 4.1. Displacement Responses in Time Series

**Figure 7.**
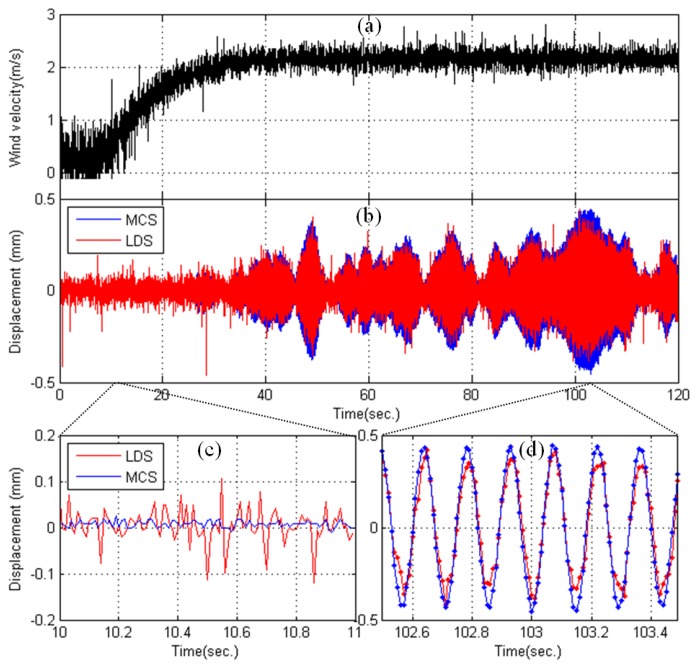
Observed test results. (**a**) Wind velocity; (**b**) Displacement responses (MCS *vs*. LDS); (**c**) Displacement responses from 10 s to 11 s; (**d**) Displacement responses from 102.5 s to 103.5 s.

Figure 7 presents the test results recorded during the wind tunnel tests. For this response measurement, the wind loads were set at a constant 2.2 m/s, and the rotation velocity was set at 100 rpm (Figure 7a). Figure 7b compares the displacement data obtained using the MCS with those obtained using the LDS. A displacement measurement MCS represents the values of the marker movements (Y-direction) obtained from the motion-capture camera installed at the top of the pylon (M1), and the LDS measurements represent the values of the movement of the highest point, which was obtained from the LDS (L1). The figure indicates that the displacement obtained from the MCS was slightly larger than that obtained using the LDS. During the measurement time of 120 s, the differences in the maximum values were 0.08 mm (MCC: 0.44 mm, LDS: 0.36 mm) in the positive direction and 0.12 mm (MCC: −0.45, LDS: −0.33) in the negative direction. Considering that the accuracy of the two measurement systems is approximately 0.1 mm, the difference in the positive direction between the two systems was within the allowable limits of error. However, the error in the negative direction was 0.12 mm, which is beyond the allowable limits of error.

The difference in the time-domain data between the two measuring systems is thought to be due to the slight difference between the axes of the MCS and the axes measured by the LDS. In other words, the global and local axes are matched using the calibration and preliminary measurement results of the static condition of the target structure in the MCS, but the measuring direction must be judged with the naked eye when installing the sensors for the LDS, which may explain the slight error in the local axis of the target object. Furthermore, comparing the noise levels between the MCS and LDS over 1 s (10 s ∼ 11 s) in Figure 7c, the maximum value in the MCS was 0.02 mm, and the maximum value in the LDS was 0.11 mm, which is a difference of approximately five times. Figure 7d also shows that the observed displacement from the MCS is more stable and smoother than that obtained from the LDS, which showed a ragged shape in its peaks. Thus, the test results of the MCS, which established axes through accurate measurements, appear to be more accurate than those of the existing measurement equipment (LDS).

### 4.2. System Identification

We computed the dynamic properties (natural frequency, damping ratio, and mode shape) of the test model using displacement data to compare the performance level of the displacement measurements by the MCS and LDS. The system identification (SI) method, which was used to discover the dynamic properties in this study, is a frequency domain decomposition (FDD) method [35]. This method separates noise and extracts dynamic properties while separating the cross-power spectrum on the time-series data of each measured point using the singular value decomposition (SVD) method. The MCS uses a three-degrees-of-freedom displacement (x-, y-, and z-axial displacements) measured at seven markers from the top of the specimen. However, only displacement data in the y-direction are available using the LDS (L1, L3, and L5 in Figure 6) because the displacement in the x-axis (strong axis) contains too much noise to use the SI method.

#### 4.2.1. Natural Frequency

**Figure 8.**
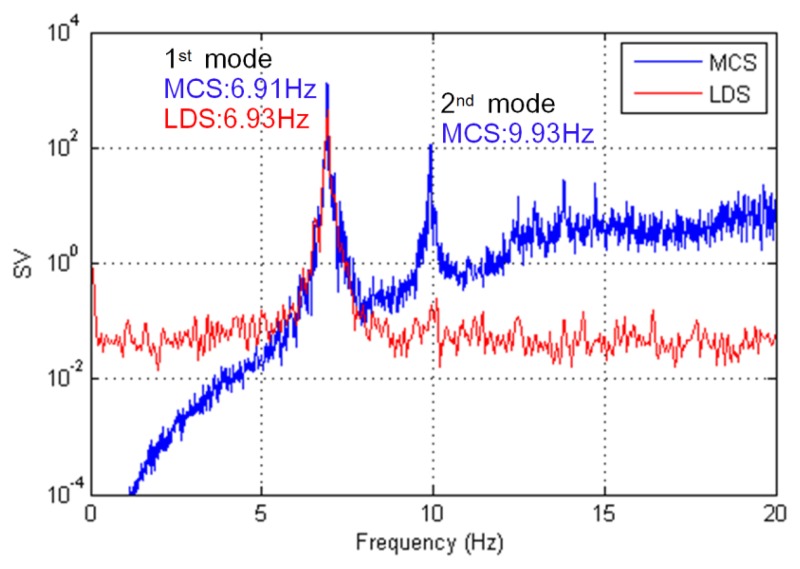
Extraction of the natural frequency.

A singular value (SV) plot was created using the FDD method for each measurement system, as shown in Figure 8. The natural frequencies for the MCS and LDS measurements were determined using the peak points in the SV plot. In the case of the MCS, we could extract the first mode (y-axis, 6.91 Hz) and the second mode (x-axis, 9.93 Hz) of the natural frequency. In the case of the LDS, only the first mode (y-axis, 6.93 Hz) of the natural frequency was extracted because the displacement in the x-axis representing the second mode could not be used due to too much noise. The two methods provided similar results for the natural frequency on the y-axis.

#### 4.2.2. Damping Coefficient

**Figure 9.**
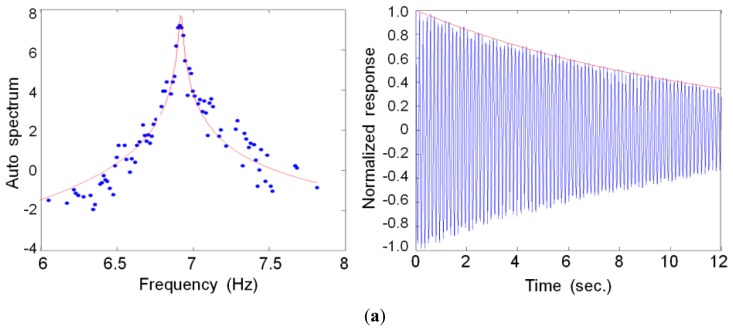

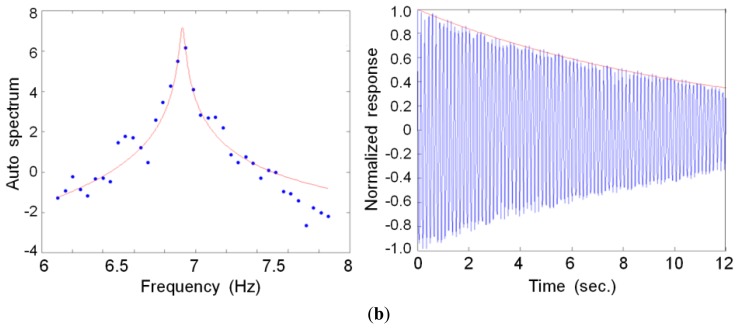
Auto spectrum and free vibration wave for extracting the damping value (1st mode). (**a**) MCS; (**b**) LDS.

In the SV plot, each mode was separated by a one-degree-of-freedom system using the modal assurance criteria (MAC), followed by conversion into the time domain, as shown in Figure 9. We estimated the damping value of the specimen by applying the logarithmic decrement method to the gained free-vibration waves. The first (0.203%) and second (0.199%) modal damping constants were calculated for the MCS, whereas damping for only the first (0.359%) mode was extracted for the LDS measurements. In the first mode, the ratio of the MCS (0.203%) to LDS (0.359%) measurements indicated a 177% difference. However, because this difference was at a much lower damping value than the general damping values, this discrepancy was likely not the result of errors arising from these two measurement methods.

#### 4.2.3. Mode Shape

**Figure 10.**
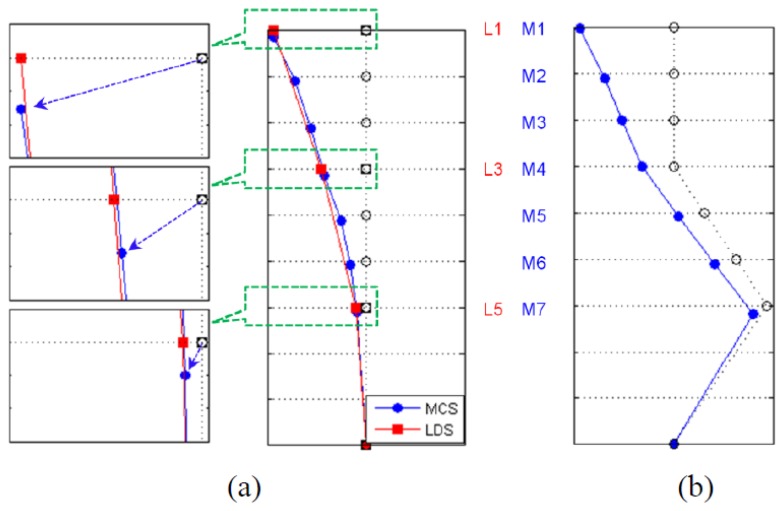
Mode shape. (**a**) 1st mode shape (y-axis); (**b**) 2nd mode shape (x-axis).

The mode shape could be determined from a singular vector that consists of peak points in the SV plot; the extraction results of each measurement method are shown in Figure 10. For the MCS, the first and second mode shapes were extracted about the y- and x-axes of the specimen. For the LDS, the second mode (x-axis) could not be extracted because only the displacement data from L1, L3, and L5 were used to perform the SI. To compare the characteristics of the extraction results of each measurement method, we provide a comparison of the first-mode shapes in Figure 10a. In the case of the MCS, not only the y-axis mode (deformation in the horizontal direction) but also the z-axis mode (deformation in the vertical direction) values could be shown for the first mode because the mode information about the three degrees-of-freedom (x, y, and z translation) could be extracted. This result confirmed that the MCS could efficiently extract the bending deflection mode shape by collecting the 3D coordinate information of the structure. In contrast, three sensors are needed when attempting to measure the displacements in three axes (*i.e.*, x-, y- and z-axes) using existing measurement devices, which results in a large number of sensors and a complex wired system as the number of measuring points increases. Installing sensors and measuring the deformation in the vertical direction with conventional measurement devices were especially difficult. Therefore, this result also showed the efficiency of the MCS, which could simultaneously measure the three-axis behaviors of the structure from a marker when applied to a wind tunnel test in which the measuring instruments can affect the wind flow and structural performance.

## 5. Conclusions

In this paper, we have proposed a remote sensing system to measure position using motion-capture cameras for wind tunnel tests used to measure the deformation of a target structure subjected to a wind load. This system, which overcomes the limitations of the existing sensors (*i.e.*, sensor effects, measurement frame for installing sensors, and wiring on the wind flow), can estimate the 3D coordinates using two-dimensional information. Specifically, the test equipment can be arranged to minimize the effects on the flow of the simulated wind during the wind tunnel tests. We obtained the following results by comparing the measurements of the proposed system with those of the existing measuring systems (LDSs).

A comparison between the displacement obtained from the MCS and that obtained using the LDS indicated that the displacement of the former was slightly greater than the displacement of the latter. The difference in the amplitude of the time-history data was thought to be caused by the axis set in the LDS, which did not match the axis measured by the MCS. In other words, the global axis and the local axis were set to match using the calibration and preliminary measurement results of the static condition of the target structure with the MCS. However, we necessarily judged the measurement direction by a simple visual assessment while installing sensors for the LDS, which was thought to explain the slight error in the local axis of the target object. Thus, the test results of the MCS, which set up axes using an accurate measurement, appear to be more accurate than those from the existing measurement equipment.

Moreover, to determine the dynamic properties of structures, we demonstrated the possibility of calculating accurate values of the natural frequency, damping constant, and mode shape. With respect to the results of the mode shape extraction in the MCS, the y- and z-axis mode values could be shown for the first mode because mode information about three degrees-of-freedom (x, y, and z-translation) could be extracted. In this experiment, the MCS could extract the bending deflection mode shape by simultaneously collecting the 3D coordinate information of the structure.

The results described above indicate that the remote sensing system to measure position using motion capture proposed in this study can minimize the effect on wind flow, which is a limitation of existing measurement equipment or wired measurement system for wind tunnel tests. In addition, the proposed system can perform the roles of existing measurement equipment and provide more accurate 3D measurements. Finally, installing the MCS as a built-in measurement system in a wind tunnel test facility, such as the HFBB and the SM-PSS, is possible when considering a long measurement range and a broad camera angle, although the current cost of the MCS is relatively high compared with that of existing measurement devices.
